# Advanced Cellular Models for Drug Testing and Discovery to Target Cardiac Fibrosis Progression

**DOI:** 10.3390/ijms27219729

**Published:** 2026-10-30

**Authors:** Francesca Icolaro, Isotta Chimenti, Claudia Cozzolino, Antonella Bordin, Virginia Pontecorvi, Giacomo Frati, Elena De Falco, Erica Floris, Vittorio Picchio, Francesca Pagano

**Affiliations:** 1 Department of Medical and Surgical Sciences and Biotechnologies, Sapienza University of Rome, 04100 Latina, Italy; 2 IRCCS Maria Cecilia Hospital, GVM Care & Research, 48033 Cotignola, Italy; 3 IRCCS Neuromed, 86077 Pozzilli, Italy; 4 Institute of Biochemistry and Cell Biology, National Council of Research (IBBC-CNR), 00015 Monterotondo, Italy

**Keywords:** cardiac fibrosis, three-dimensional models, drug screening, mechanobiology, mechanotransduction, engineered heart tissue, organ-on-a-chip, antifibrotic therapy

## Abstract

Cardiac fibrosis is a complex and dynamic pathological process, central for cardiac repair, characterised by continuous cell–cell and cell–matrix interactions. Three-dimensional (3D) models are gaining increasing relevance, compared with traditional two-dimensional (2D) cellular setups, due to their ability to recapitulate key features of the fibrotic microenvironment at different levels of complexity, including cellular heterogeneity, spatial organisation and tissue mechanical properties. These systems have enabled a deeper investigation of fibrosis mechanobiology and the mechanosensitive pathways involved in disease progression, contributing to the identification of potential therapeutic targets for cardiac repair. Increasing biological complexity can reveal therapeutic responses that remain undetected in conventional models; however, the predictive value of 3D systems has so far been confirmed in vivo only for a limited number of platforms and compounds. The role of 3D models is therefore expanding beyond disease modelling towards platforms for the screening and evaluation of antifibrotic strategies, pointing to increasingly functional cardiac reparative strategies. Here, we distinguish model complexity, biological fidelity and demonstrated predictive value, compare current platforms according to their validation level and limitations, and discuss the benchmarks and standards required for their routine use in antifibrotic drug discovery. From this perspective, the development of systems that are not necessarily more sophisticated, but progressively more informative and predictive, may help bridge the gap between conventional 2D in vitro models and animal studies, ultimately improving the translational relevance of preclinical evaluation.

## 1. Introduction

The evolution of our understanding of fibrosis and the way we perceive it is intriguingly reflected in its terminology. The term fibrosis shares its etymological root with fibre, recalling the fibrous component that, long before its cellular and molecular basis could be understood, represented the most recognizable feature of the pathological process. As early as the second half of the nineteenth century, Rudolf Virchow recognised the association between fibrous tissue and disease in his *Die Cellularpathologie* published in 1858, helping to establish the concept of fibrosis as a process closely associated with pathology.

A few decades later, in 1895, Ernst Ziegler introduced the term fibroblast to describe cells within granulation tissue responsible for producing new connective tissue during wound healing. Over time, a close connection thus emerged among three concepts: fibrous tissue, fibroblasts and tissue repair. In light of current knowledge, this association is particularly meaningful, as pathological fibrogenesis and physiological tissue repair largely share the same cellular players and numerous signalling pathways [1].

From this perspective, tissue repair provides a natural starting point for understanding how a physiological response to injury can progressively evolve into a pathological process. In multicellular organisms, tissue repair represents one of the main mechanisms through which tissues respond to injury. In adult mammals, this response predominantly relies on scar formation rather than complete tissue regeneration (restitutio ad integrum), enabling rapid wound closure and restoration of the structural integrity of the injured organ, although at the expense of fully recovering its original architecture and function [2].

Scar formation therefore represents a physiological component of the healing process, provided that it remains tightly controlled in space and time. Following acute injury, activation of resident fibroblasts, extracellular matrix (ECM) remodelling and deposition of new connective tissue contribute to restoring the integrity of the damaged tissue [1].

What distinguishes a physiological reparative response from a fibrotic one therefore lies not so much in the nature of the mechanisms involved, but in their appropriate spatiotemporal coordination and, above all, in the ability of the reparative program to resolve once tissue integrity has been restored. Conversely, persistence of the injurious stimulus or damage exceeding the organism’s reparative capacity disrupts this balance, altering the intercellular communication that governs healing and promoting a chronically active reparative microenvironment [1].

Among the different forms of organ fibrosis, cardiac fibrosis is particularly relevant from a clinical perspective. Despite their marked etiological heterogeneity, many cardiovascular diseases converge on a common process of fibrotic remodelling [3]. Cardiovascular diseases remain the leading cause of death worldwide and therefore represent one of the major challenges in modern medicine [4].

This is partly related to the limited regenerative capacity of the adult heart, due to the low proliferative potential of cardiomyocytes. Following acute injury, such as myocardial infarction, scar formation initially represents an essential adaptive response that preserves the structural integrity of the organ. However, when the reparative program persists or is continuously reactivated by chronic pathological stimuli, the progressive accumulation of fibrous tissue alters myocardial architecture, increases ventricular stiffness, impairs contractile function, promotes arrhythmogenesis and ultimately contributes to the progression towards heart failure [5].

More specifically, fibrosis has traditionally been described as the consequence of persistent fibroblast activation and excessive deposition of extracellular matrix components, particularly fibrillar collagens, fibronectin and other structural proteins [1,5].

In the cardiac context, this view was further supported by the identification of resident fibroblasts as the main cells responsible for maintaining extracellular matrix homeostasis under physiological conditions and for scar formation following injury. However, the progressive characterisation of their origin, plasticity and activation states has shown that the very concept of the cardiac fibroblast requires substantial redefinition, paving the way for a more comprehensive understanding of the fibrotic response [6].

For many years, this framework represented the dominant paradigm for investigating cardiac fibrosis and substantially contributed to the identification of the major profibrotic signalling pathways and the mechanisms governing fibroblast activation. It also provided the biological rationale for developing therapeutic strategies aimed at limiting extracellular matrix deposition or directly modulating fibroblast activity. However, knowledge accumulated over the past decade has progressively shown that, although fundamental, this interpretation mainly describes the final outcome of fibrosis without fully capturing its biological complexity. Indeed, the progression of cardiac fibrosis does not depend solely on fibroblast activation or extracellular matrix accumulation, but emerges from the dynamic interplay among multiple cellular populations, biochemical signals and biomechanical cues that collectively sustain pathological myocardial remodelling [3,7].

Within this new perspective, the role of the extracellular matrix has also been reconsidered. The ECM is no longer viewed solely as the final product of fibroblast activity or as a structural scaffold for the tissue, but is now recognised as a dynamic regulator of cellular behaviour. Through its compositional, topographical and mechanical properties, it actively modulates the proliferation, activation and phenotypic state of resident cells while also influencing further matrix deposition, thereby contributing to the maintenance of the fibrotic response [1,8].

This evolving view of fibrosis also extends to the mechanical component of the cardiac response. Interestingly, the parallelism between physiology and pathology applies not only to cellular populations and molecular mechanisms, but also to mechanical forces. Mechanical forces are an integral part of normal myocardial physiology and cardiac fibroblasts are continuously exposed to the stresses generated by the contractile activity of the heart while largely maintaining a quiescent phenotype. Following injury, however, changes in the mechanical microenvironment, together with the biochemical and structural signals associated with the tissue response, contribute to fibrotic remodelling [9,10]. In this context, mechanosensing becomes particularly relevant, as it allows fibroblasts to perceive and interpret the physical characteristics of their surrounding microenvironment [10,11].

For all the above, cardiac fibrosis should no longer be viewed as the simple sum of independent pathological events, but rather as a highly organised and dynamic pathological microenvironment in which cellular and non-cellular components interact with biochemical and mechanical signals. In the recent literature on fibrotic diseases, this concept has been described as the fibrogenic niche: a tissue microenvironment in which cellular and non-cellular components interact to initiate, sustain and amplify fibroblast activation and disease progression [12].

This redefinition of cardiac fibrosis has changed not only how its progression is studied, but also how new therapeutic strategies are conceived. For many years, drug discovery focused predominantly on the modulation of individual molecular pathways or specific cellular targets. This reductionist approach enabled the identification of numerous therapeutic targets and supported the development of a growing range of pharmacological strategies, including small molecules and biological or RNA-based approaches [13].

The growing recognition of the mechanobiological dimension of fibrosis also has important implications for its experimental modelling. Models should account for cell–ECM interactions and for the mechanical context in which different signals are perceived and integrated [9,10].

From this perspective, the increasing use of three-dimensional models does not simply reflect the need for greater structural biomimicry. Moreover, three-dimensionality alone does not guarantee an accurate representation of the mechanical microenvironment. Rather, the relevance of a model depends on how effectively it reproduces features that are important for the biological process under investigation. The development of next-generation antifibrotic strategies therefore requires models capable of evaluating drug candidates within a biological context that reproduces key aspects of the fibrotic microenvironment, with the aim of improving their predictive performance in preclinical studies [13,14].

On these grounds, this review examines three-dimensional models of cardiac fibrosis in light of the growing understanding of its mechanobiological regulation. Particular attention is given to how these platforms reproduce relevant features of the fibrotic microenvironment and support the evaluation, repurposing and discovery of antifibrotic pharmacological strategies.

We previously reviewed multicellular 3D models as tools to study the cellular and molecular mechanisms of cardiac fibrosis [15]. Here, we focus on how 3D platforms should be selected, validated and interpreted when they are used to evaluate antifibrotic drugs. To this end, we distinguish model complexity, biological fidelity and demonstrated predictive value, compare available platforms by their technical features, validation level and limitations, and critically discuss how mechanobiological features should guide assay design and the interpretation of drug responses. We also outline the benchmarks and standards needed before these platforms can be routinely used in drug discovery.

This work is a narrative review, and the platforms discussed represent a selection of the available literature rather than an exhaustive survey. The literature was searched in PubMed for articles published in the last ten years, using combinations of terms related to cardiac fibrosis modelling in vitro. Original studies were extensively presented when they used a 3D in vitro model of cardiac fibrosis and tested at least one pharmacological or advanced therapeutic intervention with fibrosis-relevant readouts. Studies using only 2D cultures, only animal models, or 3D cardiac models without a fibrotic component were excluded. Among the eligible studies, we prioritised those that compared platforms or treatments directly, provided functional or biomechanical readouts, or included in vivo validation. Studies of non-cardiac fibrotic tissues were included only to illustrate mechanobiological principles or technologies not yet available in cardiac models, and are explicitly identified as such.

## 2. Mimicking Cardiac Fibrosis: 3D Models as Platforms for Evaluating Antifibrotic Compounds

Cardiac fibrosis represents one of the pathological processes in which three-dimensional models have proven particularly useful. Owing to its marked complexity, it requires sophisticated biomimetic systems capable of faithfully recapitulating not only cellular features, but also multicellular interactions, extracellular matrix remodelling as well as the mechanobiological components of the microenvironment.

The growing use of three-dimensional models in the search for novel cardiac antifibrotic therapies has driven their progressive evolution towards increasingly sophisticated biomimetic platforms [15].

Multicellular three-dimensional models, initially used as complex endpoint platforms to validate the effect of existing drugs belonging to different therapeutic classes, are now powerful tools providing functional and mechanobiological information for comparing the mechanism of action of antifibrotic candidates and selecting those with the greatest translational potential.

The following sections will present a selection of such models with different degrees of complexity, employed to investigate both drugs already used in cardiovascular diseases and emerging therapeutic approaches, including RNA-based strategies.

Before discussing individual platforms, it is useful to distinguish three properties that are often used interchangeably. “Model complexity” refers to the number of components integrated in a platform (cell types, matrix, mechanical or electrical stimulation, perfusion, sensors). “Biological fidelity” refers to how closely the model reproduces the specific features of human cardiac fibrosis that are relevant to the question under study, such as the fibroblast activation state, matrix composition and stiffness, or the impact of fibrosis on cardiomyocyte function. “Predictive value” is the demonstrated ability of a model to anticipate the response to a treatment in an independent, more relevant system (in vivo models or, ultimately, patients). These properties are related but not equivalent. Greater complexity can increase biological fidelity, but it can also add variability and reduce throughput. Biological fidelity is necessary but not sufficient for predictive value, which can be established only through validation against external outcomes. To make these differences explicit, we propose a practical three-level framework for the studies discussed in this review, intended as a tool for comparison rather than as an established classification. Level 1 corresponds to characterisation based only on internal in vitro readouts; Level 2, to benchmarking against reference compounds and/or direct comparison with 2D cultures within the same study; and Level 3, to in vivo confirmation of model-derived predictions in an independent animal model. Level 3 indicates concordance with animal models and should not be equated with validated clinical predictive performance, which has not yet been demonstrated for any of the platforms discussed. The level reached by each platform, with a brief justification, is reported in Table 1: most platforms have reached Level 2, and only a few have reached Level 3.

### 2.1. Three-Dimensional Models for Comparative Drug Evaluation

An example of an emerging therapeutic approach is represented by a human cardiac fibrosis-on-a-chip (hCF-on-a-chip) platform developed to evaluate the response to drugs with different therapeutic rationales in a microenvironment that more faithfully reproduces cardiac fibrosis [16]. This platform was generated by co-culturing human cardiac fibroblasts with hiPSC-derived cardiomyocytes and was able to recapitulate several hallmarks of cardiac fibrosis, including increased extracellular matrix deposition, enhanced tissue stiffness, impaired contractile function and elevated BNP secretion. To assess its potential as a platform for the preclinical evaluation of antifibrotic therapies, it was employed to compare the effects of three drugs widely used in clinical practice with distinct mechanisms of action: losartan, which limits profibrotic signalling by blocking angiotensin II-mediated TGF-β activation; carvedilol, a beta-blocker with adjunctive antioxidant and anti-remodelling effects used in heart failure management; and pirfenidone, an antifibrotic agent approved for idiopathic pulmonary fibrosis that directly targets TGF-β-dependent myofibroblast differentiation and collagen synthesis. While losartan and carvedilol are widely used for their established benefits on cardiac remodelling, neither was specifically designed to target fibrosis and their effects on the fibrotic phenotype remain largely indirect. In contrast, pirfenidone has emerged as a reference compound for benchmarking newly developed antifibrotic therapies in preclinical cardiac models, although it is not approved for cardiac fibrosis, and its mechanisms of action in cardiac fibrosis have been extensively reviewed [17]. Accordingly, the analysis of multiple functional and molecular parameters in this model indicated that pirfenidone outperformed the other two compounds, exhibiting the most comprehensive antifibrotic profile and improving not only molecular biomarkers but also the biomechanical and functional properties of the engineered tissue. These effects were assessed through a multiparametric panel of readouts, including collagen deposition, tissue stiffness, passive tension, force of contraction, excitation threshold, BNP secretion and fibrosis-associated transcriptomic and microRNA signatures.

Pirfenidone was also evaluated in a different 3D platform, an electrospun nanofibrous yarn scaffold colonised by cardiac fibroblasts only, where it was compared with honokiol, a natural compound with previously reported antifibrotic and anti-remodelling activity [18]. In this system, pirfenidone reduced fibrotic parameters but was less effective than honokiol [19]. The two studies differ in cell composition, scaffold, fibrosis induction and endpoints, and a compound may perform differently simply because each platform captures different aspects of the disease.

Once the ability of three-dimensional platforms to discriminate between therapeutic candidates with different degrees of antifibrotic efficacy had been demonstrated, the next logical step was to extend their application to the screening of compound libraries.

For example, a contractile 3D myocardial tissue composed of primary neonatal rat cardiomyocytes, cardiac fibroblasts and bone marrow-derived macrophages in a collagen hydrogel, subjected to oxygen–glucose deprivation, was used to screen 76 compounds isolated from *Toxicodendron vernicifluum*. Tissue contraction, quantified by image analysis, and cell proliferation were used as readouts. The same library was screened in parallel in a conventional 2D model of TGF-β-stimulated cardiac fibroblasts. The two platforms gave only partially overlapping results: 10 compounds were active in both models, whereas 22 gave discordant results (10 active only in 2D and 12 only in 3D). Three structurally related compounds representative of the different groups were then tested in mice after coronary artery ligation. The two compounds active in the 3D model, LQ-40 and SQ-3, improved cardiac function and reduced fibrosis, whereas LQ-39, active only in the 2D model, did not [20]. The authors attributed this difference, at least in part, to effects on macrophages, a cell type absent from the 2D assay. This study is one of the few that directly compares 2D- and 3D-based compound selection against in vivo outcomes (validation Level 3). However, the in vivo validation was limited to three compounds of the same chemical class, and the model was built with rodent cells. These results therefore support, but do not prove, a general advantage of 3D screening, and cannot be extended to other 3D platforms without similar validation.

Collectively, these studies show that 3D platforms can produce compound rankings that differ from those obtained in 2D. In one case, the 3D-based selection was more consistent with in vivo efficacy [20]. Whether 3D models generally reduce false-positive and false-negative rates remains to be shown in larger, prospectively designed comparisons.

More generally, the drug responses described in this section were obtained in platforms that differ widely, and several platform-specific factors can shape, and potentially bias, the outcome. The fibrosis trigger can drive specific readouts: exogenous TGF-β produces a strong, synchronous activation that favours compounds acting on the TGF-β axis itself, whereas hypoxia, cyclic stretch or an increased fibroblast-to-cardiomyocyte ratio activate partially different pathways converging on the same cellular response directly driven by TGF-β signalling. In addition, cell composition and origin may capture only part of the response: fibroblast monocultures cannot detect effects mediated by cardiomyocytes or macrophages (as shown for compounds acting through macrophages [20]); primary, immortalised and iPSC-derived cells differ in their baseline activation and species. Regarding matrix and mechanics, the stiffness and composition of the scaffold set the baseline mechanical activation of fibroblasts and can mask or amplify the effects of drugs that act on mechanosensitive pathways (see Section 3.2). Finally, molecular markers, matrix deposition, tissue mechanics and contractile function do not always change together, and concentrations are rarely matched to clinically relevant exposure. For these reasons, results obtained in different platforms are best compared qualitatively. A quantitative ranking of compounds requires testing them side by side, under identical conditions, against shared reference compounds (see Section 4.3).

### 2.2. Biomimetic Platforms for Advanced Therapeutic Strategies

The evolution of biomimetic three-dimensional models has not only improved the ability to recapitulate the structural and mechanobiological features of cardiac fibrosis development, but has also highlighted how therapeutic responses are strongly influenced by the surrounding microenvironment. By faithfully reproducing tissue architecture, extracellular matrix composition and biomechanical stimuli, these models enable the evaluation of therapeutic efficacy under conditions that are more representative of the human disease. In this context, increasing biomimetic complexity has been shown not merely to confirm the results obtained with conventional models but also to profoundly modify them, revealing mechanisms of action and differences in therapeutic efficacy that remain undetectable in two-dimensional systems.

For example, one study investigated a combination of four cardiogenic microRNAs (miR-1, miR-133, miR-208 and miR-499), hereafter referred to as miRcombo, which had previously been shown to directly reprogram cardiac fibroblasts into cardiomyocyte-like cells both in vitro and in vivo [21], although with limited efficiency in vivo. The authors therefore explored its therapeutic potential using a hydrogel-based engineered cardiac patch, a three-dimensional tissue construct designed to mimic the structural and extracellular matrix features of the native myocardium.

The use of this platform revealed a close correlation between the cellular reprogramming induced by the microRNA cocktail and the expression of matrix metalloproteinases (MMPs). Within the same study, MMP production was compared between conventional two-dimensional cultures and this specific three-dimensional model. This comparison demonstrated that MMP expression was significantly higher in the three-dimensional platform. Interestingly, when an MMP inhibitor was administered in the same model together with the microRNA cocktail, the therapeutic effect of the latter was significantly reduced. Therefore, this platform proved not only effective in validating an advanced therapeutic strategy such as the miRcombo in a more physiologically relevant preclinical model but also in uncovering a mechanism that had remained largely unexplored because of the intrinsic limitations of conventional two-dimensional systems [22].

A further step in this direction was achieved by evaluating miRcombo in a platform that also incorporates cyclic mechanical stimulation [23]. In this study, the authors developed uScar, a miniaturised biomimetic three-dimensional model of human cardiac fibrosis in which human atrial cardiac fibroblasts (AHCFs), embedded within fibrin-based microtissues, were exposed to 10% uniaxial cyclic stretch at 1 Hz to induce a fibrotic phenotype. The platform was initially validated by assessing the antifibrotic activity of two clinically available compounds, pirfenidone and tranilast, an anti-allergic drug that suppresses TGF-β expression and activity and has shown efficacy in animal models of cardiomyopathy [24]. Both compounds effectively attenuated myofibroblast activation and extracellular matrix deposition, confirming the suitability of the platform for therapeutic testing. It was subsequently employed to evaluate the more innovative therapeutic strategy based on miRcombo. In contrast to the findings of the previous study, this more dynamic model attenuated the antifibrotic effects of the treatment, highlighting the importance of reproducing the mechanobiological aspects of the disease, which represent a key determinant of the therapeutic efficacy of the tested strategies.

### 2.3. Next Generation of Biomimetic Platforms: Beyond Drug Screening

Although the application of three-dimensional models for evaluating innovative therapeutic strategies is still relatively limited in the field of cardiac fibrosis, these approaches have already reached an advanced level of development in other fibrotic diseases, such as liver fibrosis, where more complex biomimetic platforms have been used not only to assess therapeutic efficacy but also to uncover key mechanistic information [25].

The experience gained in other fibrotic diseases therefore suggests an evolutionary trajectory that could progressively be extended to cardiac fibrosis models, fostering the development of increasingly biomimetic platforms that more faithfully recapitulate human pathophysiology.

Building on this evolution, recent studies have highlighted that the next generation of three-dimensional cardiac fibrosis models should move beyond the mere recapitulation of the histopathological features of the disease by progressively integrating cellular, biomechanical and functional components that are increasingly representative of human tissue. From this perspective, the goal is not only to reproduce the fibrotic microenvironment but also to develop platforms capable of providing reliable information for the preclinical evaluation of novel therapeutic strategies [26].

Among the most promising platforms developed in recent years, Biowire II, a biomimetic engineered heart tissue platform designed to generate long-term cultured cylindrical cardiac tissues resembling human trabeculae [27], illustrates the ongoing evolution of three-dimensional models toward more informative preclinical platforms. Its ability to recreate a physiologically relevant cardiac microenvironment, while enabling label-free structural and functional assessment of tissue remodelling, allows the platform to faithfully reproduce key hallmarks of human cardiac fibrosis. To evaluate its potential for drug screening, PCI (p-guanidinomethyl-phenylacetyl-Arg-Val-Arg-4-amidinobenzylamide), a polyarginine furin inhibitor that prevents the proteolytic activation of profibrotic mediators such as TGF-β, was administered both during the early stages of fibrotic phenotype development and at a later stage, when pathological remodelling had already been established. Whereas early treatment resulted in a marked reduction in collagen deposition, accompanied by improved biomechanical properties of the tissue, late administration produced a more limited benefit, mainly reflected by a reduction in passive tension. These findings remain a proof of concept (validation Level 1): the stage-dependent effect of furin inhibition was not tested in vivo, and the optimal dose, timing and mechanism remain to be defined. Nevertheless, the possibility of comparing the same treatment at different stages of disease progression shows how 3D platforms can address questions, such as the therapeutic window, that endpoint assays in 2D cannot [28].

The potential of these platforms is further illustrated by a recent multiscale drug discovery strategy, in which high-throughput phenotypic screening performed on human iPSC-derived cardiac fibroblasts was followed by progressive validation in experimental models of increasing complexity, including human-engineered heart tissues (EHTs) and two murine models of cardiac fibrosis, induced by pressure overload (transverse aortic constriction) and by ischaemia–reperfusion. This approach led to the identification of artesunate, a semisynthetic antimalarial drug repurposed for its antifibrotic potential, together with MD2 as its molecular target, highlighting the ability of integrated screening pipelines not only to prioritise candidate therapeutics but also to uncover their underlying mechanisms of action. In this pipeline, the 3D engineered heart tissue served as an intermediate confirmation step between high-throughput screening and in vivo testing, and the prediction was confirmed in two independent mouse models (Level 3) [29]. Notably, the 3D tissue was used to confirm rather than to discover the hit, which illustrates a realistic current role for complex models in drug discovery.

Taken together, these examples (Table 1) show that 3D models are being used at different stages of antifibrotic drug development, from the comparison of known compounds to stage-dependent testing and integrated screening pipelines. However, they also show that the level of validation varies widely: most platforms have been characterised only through in vitro readouts and comparisons with reference drugs (Levels 1–2), and only a few studies have confirmed model-derived predictions in vivo (Level 3) [20,29,30]. Integrating more biomimetic structural, biomechanical and functional features may improve the reliability of preclinical evaluation, but only if accompanied by appropriate validation.

### 2.4. Selecting a Model for Antifibrotic Drug Testing

No single platform is best for all purposes, and the appropriate model depends on the question asked and on the type of fibrosis targeted (Table 1). As an example, replacement (post-infarction) fibrosis develops after cardiomyocyte loss and involves ischaemia, inflammation, a sharp border zone and phase-dependent remodelling. It is best modelled by platforms that include an ischaemic or local injury and immune or multiple cardiac cell types, such as hypoxia/oxygen–glucose deprivation in multicellular tissues [20], localised cryoinjury in engineered heart tissues [30] or focal “heteropolar” tissues [28]. Such models allow drug effects to be tested at different phases of repair. By contrast, diffuse interstitial fibrosis, typical of pressure overload, hypertensive heart disease, HFpEF and cardiomyopathies, develops progressively within viable myocardium. It is better reproduced by models in which fibrosis affects the whole tissue and its function, such as TGF-β-stimulated or fibroblast-enriched engineered tissues [16,28], or mechanically loaded constructs [23]. Here, mechanical and diastolic endpoints such as passive tension are particularly relevant.

The stage of drug development also matters. Simple, high-throughput formats (2D or simple 3D fibroblast assays) remain the most efficient for primary screening of large libraries. Intermediate-complexity 3D models with compact readouts (e.g., contraction-based assays in 96-well format [20,30]) are suited to secondary screening and to resolving discordant hits. The most complex, low-throughput platforms, which include cardiomyocytes, mechanical conditioning and functional readouts [16,28,29], are best used for confirming leads, studying mechanism of action and defining the therapeutic window, before in vivo validation. Finally, cell composition should match the expected mechanism of action. Fibroblast monocultures are adequate for compounds that act directly on fibroblast activation, but they will miss compounds that act through macrophages [20] or cardiomyocytes, as well as cardiotoxic effects. These recommendations are summarised in Table 2, which links disease context, drug mechanism, required cell types and recommended endpoints.

**Table 1:** Comparative overview of 3D platforms used for antifibrotic drug evaluation.

3D Model/Platform [Ref.]	Cell Sources	Key Biomimetic Feature	Fibrosis Induction; Mechanical Stimulation	Disease Context	Therapeutic Strategy	Main Readouts	Key Information Gained from the 3D Model	Main Limitations	Validation Level (Justification)
Human cardiac fibrosis-on-a-chip (hCF-on-a-chip) [16]	Human ventricular cardiac fibroblasts and hiPSC-derived cardiomyocytes	ECM remodelling and altered tissue mechanics in a contractile 3D cardiac microtissue	TGF-β1-dependent; no external stretch	Interstitial cardiac fibrosis/heart failure	Comparative evaluation of losartan, carvedilol and pirfenidone	Collagen deposition; tissue stiffness/passive tension; contractile force; BNP secretion; transcriptomic and miRNA signatures	Identified pirfenidone as the most effective treatment across molecular, biomechanical and functional readouts	Limited cellular complexity (no immune or vascular cells); immature hiPSC-CMs; exogenous TGF-β1-dependent fibrosis induction; no in vivo validation	Level 2: comparison of three clinically used drugs, including the reference compound pirfenidone; no in vivo confirmation
Biomimetic electrospun yarn scaffold [19]	Primary neonatal mouse cardiac fibroblasts	Fibrous 3D architecture mimicking cardiac ECM organisation and cell–matrix interactions	TGF-β1-dependent; static	Interstitial fibrosis (fibroblast activation)	Comparative evaluation of honokiol and pirfenidone	Collagen/ECM deposition; fibrotic markers; tensile modulus	Honokiol showed greater antifibrotic efficacy than pirfenidone across molecular and biomechanical endpoints	Fibroblast monoculture: no functional cardiac readout; synthetic scaffold; no in vivo validation	Level 2: pirfenidone used as reference comparator; no in vivo confirmation
Collagen hydrogel-based platform [20]	Primary neonatal rat cardiomyocytes and cardiac fibroblasts; rat bone marrow-derived macrophages	Collagen-based 3D microenvironment supporting multicellular interactions under hypoxic conditions	TGF-β1-independent (oxygen–glucose deprivation); static	Ischaemic/post-infarction	Screening of 76 *T. vernicifluum*-derived compounds	Tissue contraction; cell proliferation; cytotoxicity	3D-selected LQ-40 and SQ-3 showed efficacy in vivo, unlike the 2D-selected LQ-39	Rodent-derived primary cells; short culture duration; contraction-based phenotypic screening; in vivo validation limited to three selected compounds	Level 3: parallel 2D vs. 3D screening; 3D-selected hits confirmed in LAD-ligated mice
Hydrogel-based engineered cardiac patch [22]	Neonatal mouse cardiac fibroblasts	Fibrin-based 3D microenvironment supporting cell–matrix interactions and tissue remodelling	3D fibrin hydrogel; no fibrotic stimulus; static	Reprogramming (scar-replacement strategy)	miRcombo (miR-1, miR-133, miR-208 and miR-499)	Cardiac gene/protein expression; reprogramming efficiency; MMP expression/activity	Enhanced miRcombo-mediated cardiac reprogramming and revealed an MMP-dependent mechanism	Murine cells; no fibrotic induction; reprogramming rather than antifibrotic endpoints	Level 2: direct 2D vs. 3D comparison; 3D-derived findings not tested in vivo
uScar [23]	Human atrial cardiac fibroblasts	Fibrin-based microtissues subjected to controlled cyclic mechanical strain	TGF-β1-independent (cyclic mechanical strain); 10% cyclic uniaxial strain at 1 Hz	Mechanically driven interstitial fibrosis	Pirfenidone, tranilast and miRcombo	α-SMA; Collagen I/Fibronectin deposition; cardiomyocyte reprogramming markers	Mechanical stimulation reduced miRcombo efficacy, revealing a strong dependence of therapeutic response on the mechanical microenvironment	Fibroblasts only (no CMs); atrial origin; single donor; no in vivo validation	Level 2: reference drugs (pirfenidone, tranilast) and comparison with 2D data; no in vivo confirmation
Biowire II [28]	Human iPSC-derived cardiomyocytes and human ventricular cardiac fibroblasts	Electrically conditioned cardiac tissues recapitulating fibrosis-associated mechanical and functional alterations	TGF-β1-independent (increased fibroblast content); electrical conditioning	Interstitial and focal (border-zone) fibrosis	PCI furin inhibition, tested at early vs. late stages	Passive tension; collagen deposition; active force; electrophysiological parameters	Revealed a stage-dependent therapeutic response, with earlier furin inhibition providing greater antifibrotic benefit	Immature hiPSC-CMs; no immune/vascular cells; single compound; no in vivo validation	Level 1: internal functional readouts with a single compound; no reference drug or in vivo confirmation
Human 3D engineered heart tissue (EHT) [29]	Human iPSC-derived cardiomyocytes and cardiac fibroblasts	Engineered cardiac tissue recapitulating fibrosis-associated contractile dysfunction	TGF-β1-induced fibrosis; intrinsic mechanical tension (no externally applied stretch)	Interstitial fibrosis in vitro; pressure-overload and post-infarction fibrosis in vivo	Artesunate repurposing	Contraction and relaxation velocity	Confirmed functional rescue by artesunate, supporting its progression from cell-based screening to in vivo validation	Hit discovered in 2D; 3D tier used for confirmation only; resource-intensive	Level 3: hit confirmed in two independent mouse models (TAC and ischaemia–reperfusion)
hEHT injury–repair model [30]	hPSC-derived CMs, CFs, epicardial and endothelial cells	Engineered cardiac tissue recapitulating the sequential phases of myocardial injury, fibrotic remodelling and electrophysiological dysfunction	Localised cryoinjury; fibrin–Matrigel; phased remodelling	Post-infarction focal scar	17 pathway modulators; sequential Y27632 → CHIR99021 → SB431542	Calcium propagation; fibrotic area; scRNA-seq	Identified a sequential therapeutic strategy (Y27632, CHIR99021, SB431542) that improved cardiac function and reduced fibrosis, with in vivo validation	Cryoinjury does not replicate ischaemia; immature cells; Matrigel batch variability; no immune cells	Level 3: sequential treatment confirmed in a mouse model of myocardial infarction

Abbreviations: α-SMA, α-smooth muscle actin; BNP, B-type natriuretic peptide; CF, cardiac fibroblast; CM, cardiomyocyte; ECM, extracellular matrix; EHT, engineered heart tissue; hEHT, human-engineered heart tissue; hiPSC/iPSC, human-induced pluripotent stem cell; hPSC, human pluripotent stem cell; MMP, matrix metalloproteinase. LAD, left anterior descending coronary artery; TAC, transverse aortic constriction. Validation levels follow the practical framework proposed in this review (Section 2): Level 1, internal in vitro readouts only; Level 2, benchmarking against reference compounds and/or direct 2D vs. 3D comparison; Level 3, in vivo confirmation of model-derived predictions in an independent animal model. Level 3 does not imply validated clinical predictive performance, which has not been demonstrated for any platform.

**Table 2:** Decision matrix for selecting 3D models for antifibrotic drug testing.

Disease Context/Question	Drug Mechanism	Minimum Cell Types	Recommended Model Features	Recommended Endpoints	Examples [Ref.]
Post-infarction (replacement) fibrosis	Anti-inflammatory or immunomodulatory compounds; drugs acting on early injury responses	CMs, CFs and macrophages	Hypoxia/oxygen–glucose deprivation or localised injury in a multicellular 3D tissue	Tissue contraction/compaction; collagen deposition and fibrotic area; inflammatory markers; CM viability and function	[20,30]
Post-infarction fibrosis: phase-dependent repair	Pathway modulators given at defined repair phases; sequential or combination therapies	CMs and CFs; ideally epicardial and endothelial cells	Localised injury with time-resolved remodelling; treatment at defined phases	Fibrotic area; calcium propagation and conduction; contractile function; phase-specific molecular signatures	[30]
Diffuse interstitial fibrosis (pressure overload, HFpEF, cardiomyopathies)	TGF-β pathway inhibitors; ECM-targeting and other direct antifibrotic drugs	Human CMs and CFs	TGF-β stimulation or fibroblast-enriched engineered tissues; long-term culture; prevention vs. reversal design	Passive tension/stiffness; collagen deposition; relaxation (diastolic) parameters; contractile force; BNP	[16,28,29]
Mechanically driven fibrosis	Modulators of mechanotransduction (e.g., FAK, YAP/TAZ–TEAD, ROCK, Piezo1)	CFs; CMs when cardiomyocyte effects must be assessed	Tunable or dynamic matrix stiffness; cyclic stretch matched to the disease being modelled	α-SMA; collagen I/fibronectin; YAP/TAZ localisation; tissue stiffness	[23,31,32]
Fibroblast-directed screening (any context)	Compounds acting directly on fibroblast activation	CFs	3D hydrogel or scaffold in a medium-to-high-throughput format	Myofibroblast markers; ECM deposition; proliferation; cytotoxicity	[19,20]

Reference compounds (e.g., a TGF-β receptor inhibitor and pirfenidone) and cytotoxicity controls should be included in all settings (see Section 4.3). Abbreviations: α-SMA, α-smooth muscle actin; BNP, B-type natriuretic peptide; CF, cardiac fibroblast; CM, cardiomyocyte; ECM, extracellular matrix; FAK, focal adhesion kinase; HFpEF, heart failure with preserved ejection fraction; ROCK, Rho-associated protein kinase.

## 3. Mechanobiology: How Cells Generate, Sense and Respond to Mechanical Cues

The biological fidelity of three-dimensional models, and ultimately their predictive value, depends critically on their ability to reproduce the biomechanical complexity of the fibrotic microenvironment. In this context, mechanobiology describes the dynamic relationship between cells and their microenvironment and provides the interpretative key to understanding how cells sense and translate physical properties and mechanical cues, such as matrix composition, geometry and stiffness, into biochemical signals capable of modulating their behaviour. Understanding the mechanobiological mechanisms that govern cardiac fibroblast activation is therefore essential not merely as a complementary aspect of the disease, but as the biological rationale underlying the very models discussed so far. For this reason, this chapter addresses in depth the mechanotransduction pathways and reciprocal cell–matrix interactions that make biomechanical fidelity a determining factor for the reliability of preclinical antifibrotic drug testing.

To this end, it is essential to begin with the extracellular matrix (ECM), which provides the structural framework of the myocardium while actively regulating cell adhesion, migration, differentiation and survival, thereby preserving myocardial homeostasis. Under physiological conditions, the cardiac ECM undergoes continuous remodelling through a tightly controlled balance between matrix synthesis, degradation and organisation, primarily coordinated by cardiac fibroblasts through matrix metalloproteinases (MMPs) and their endogenous tissue inhibitors (TIMPs). This dynamic equilibrium maintains not only tissue architecture but also the biochemical and mechanical properties of the myocardium [3].

Following cardiac injury, this homeostatic balance is progressively disrupted. Resident fibroblasts become activated and differentiate into contractile myofibroblasts, leading to excessive deposition of fibrillar collagens, fibronectin and other ECM components. At the same time, increased collagen crosslinking, altered matrix turnover and ECM reorganisation progressively modify tissue stiffness, viscoelasticity and structural organisation, transforming the cardiac interstitium into a mechanically and biochemically altered microenvironment that promotes persistent fibroblast activation and pathological remodelling [3,33].

Rather than representing a passive consequence of fibrosis, the remodelled ECM actively regulates cellular behaviour by providing biochemical and biomechanical signals that continuously influence fibroblast activation, inflammatory responses, vascular remodelling and cardiomyocyte function. Conversely, activated fibroblasts further remodel the matrix through ECM synthesis, degradation and reorganisation, generating a dynamic interplay between cells and their surrounding microenvironment that progressively amplifies tissue remodelling. This evolving view has shifted the concept of cardiac fibrosis from excessive matrix accumulation to a disease driven by reciprocal interactions between cells and the extracellular microenvironment [33,34].

### 3.1. Mechanotransduction in Cardiac Fibrosis and Fibroblast Responses

During fibrosis, progressive changes in ECM composition, architecture and mechanics—including increased stiffness and altered matrix organisation—reshape the biomechanical landscape experienced by resident cells. The profound biomechanical alterations due to the ECM rearrangement happening during cardiac reparative processes, deeply characterise the fibrotic cardiac microenvironment and are perceived by cardiac fibroblasts through mechanotransduction. This is the process by which mechanical stimuli are converted into biochemical signals regulating gene expression, cellular behaviour and tissue remodelling.

Mechanotransduction is therefore recognised as the mechanism through which fibroblasts integrate multiple biomechanical cues from their surrounding microenvironment to generate context-dependent cellular responses [33,35].

Within the nucleus, mechanical cues regulate chromatin organisation and transcriptional activity through multiple mechanosensitive pathways. Among these, the transcriptional co-activators YAP (Yes-associated protein) and TAZ (transcriptional coactivator with PDZ-binding motif) represent central regulators of cardiac fibroblast activation. Their nuclear accumulation in response to increased matrix stiffness and cytoskeletal tension promotes TEAD-dependent transcriptional programs driving myofibroblast differentiation and extracellular matrix production. Additional pathways, including the MRTF/SRF axis and the recently described FAK–NF-Y signalling cascade, further expand the transcriptional network linking extracellular mechanics to fibroblast phenotype and function [36,37].

Notably, the role of the YAP/TAZ pathway in cardiac fibrosis has been initially explored in a self-assembling three-dimensional culture model of cardiac fibroblasts [38]. While this model confirmed the central role of YAP/TAZ pathway in the mechanotransduction underlying the fibrotic process, it also showed that, under the same mechanical stimulus, cells respond differently depending on their spatial position. In this way, the 3D model reveals a level of cellular heterogeneity that gives rise to distinct phenotypes, which cannot be captured by conventional two-dimensional cultures. Mechanosensitive ion channels, particularly Piezo1 and members of the transient receptor potential (TRP) family, provide an additional layer of mechanical sensing by converting membrane deformation into intracellular calcium signalling that regulates fibroblast proliferation, inflammation, cytoskeletal remodelling and ECM production. Together with adhesion-mediated signalling and nuclear mechanotransduction, these pathways demonstrate that cardiac fibroblasts decode the mechanical properties of their environment through the coordinated integration of complementary mechanosensing systems rather than isolated signalling cascades. Importantly, mechanotransduction is increasingly recognised as a reciprocal rather than unidirectional process. While fibroblasts continuously sense the biochemical and biomechanical properties of the ECM, they simultaneously remodel their surrounding microenvironment through collagen deposition, matrix reorganisation, crosslinking and contractile force generation [35,39]. This bidirectional interplay, recently conceptualised as mechanoreciprocity, describes tissue remodelling as a continuous dialogue in which cells reshape the ECM while the evolving matrix simultaneously reprograms cellular phenotype through persistent biomechanical signalling [34].

The ability to reproduce and control the mechanical properties of the extracellular matrix, and thereby investigate their effects on cell behaviour, makes three-dimensional models particularly suitable for studying mechanosensing. In this context, two recent studies investigated how cardiac fibroblasts respond to changes in matrix stiffness.

In the first study, primary neonatal rat cardiac fibroblasts were encapsulated in a three-dimensional collagen I–alginate hydrogel designed for dynamic modulation of matrix stiffness. In the absence of TGF-β, matrix stiffening alone promoted a myofibroblast phenotype, as indicated by increased α-SMA expression, whereas subsequent softening partially reversed this response. Mechanistically, these stiffness-dependent changes involved integrin β1 and focal adhesion signalling [31].

The transcriptional consequences of stiffness were explored in a second study using the same cell population embedded in collagen–alginate constructs with three constant stiffness levels representative of normal myocardium, an intermediate stage and advanced fibrosis. Increasing stiffness promoted fibroblast activation and YAP nuclear localisation. Transcriptomic analysis also identified a 98-gene mechanosensitive signature enriched in focal adhesion, ECM–receptor interaction and cytoskeletal organisation pathways. The relevance of this signature was supported by its dynamic regulation in fibroblasts from myocardial infarction and pressure-overload models and its upregulation in human dilated and hypertrophic cardiomyopathy datasets [32]. Together, these findings show how three-dimensional models can isolate stiffness as a mechanical determinant of fibroblast responses and trace its effects from phenotypic changes to transcriptional regulation. However, stiffness represents only one of the physical properties through which the ECM can modulate cell behaviour and functional cardiac repair.

In cardiac cells, Piezo1 mediates mechano-chemo transduction in the heart in vivo [40], and cardiac fibroblasts express functional, mechanically activated Piezo1 channels whose activation drives p38 MAPK-dependent secretion of the profibrotic cytokine IL-6 [41]. However, how matrix architecture regulates Piezo1-dependent fibrotic signalling has so far been studied in 3D only in non-cardiac systems. In human adipose-derived stem cells cultured on nanostructured 2D and 3D scaffolds designed to reproduce fibrotic matrix architecture, Piezo1-dependent collagen synthesis was modulated by fibre alignment [42]. In a skin fibrosis model, Piezo1 sustained a stiffness-responsive profibrotic feedback loop [43]. These findings suggest that matrix architecture, and not only stiffness, should be controlled in cardiac 3D models, but whether they apply to cardiac fibroblasts remains to be tested.

This mechanobiological framework has also been investigated in a more complex three-dimensional cardiac model based on a human-engineered heart tissue (hEHT). The construct was generated from human pluripotent stem cells and comprised different cardiac cell populations embedded in a fibrin–matrigel matrix. Localised cryoinjury induced a confined lesion that progressively developed into a fibrotic region, allowing post-injury remodelling to be followed over time.

Through single-cell RNA sequencing, the authors characterised the cellular heterogeneity of the construct, revealing changes in the states of different cell populations according to both the remodelling phase and the region affected by injury, with epicardial and endothelial cells also contributing to the process. In parallel, remodelling was accompanied by progressive electrophysiological alterations, characterised by slower and less coordinated calcium signal propagation within the remodelled region. Phase-dependent analysis of the remodelling process further enabled the identification of signalling pathways associated with its different stages, revealing the early involvement, among others, of TGF-β and Rho/ROCK, followed at later stages by pathways including WNT, PI3K and NF-κB.

Given its ability to reproduce several features of in vivo remodelling, the model was subsequently miniaturised and used as a platform for pharmacological screening. To this end, nine pathways identified through the analysis were selected for pharmacological screening using 17 pathway-specific modulators. Particularly relevant in this mechanosensing context, Rho/ROCK inhibition by Y27632 was able to counteract some of the remodelling -induced alterations, although recovery remained only partial. CHIR99021, an activator of WNT signalling, and SB431542, an inhibitor of TGF-β signalling, also showed more pronounced effects than the other modulators tested. After assessing the effects of each compound individually and considering the different pathways involved at each stage of remodelling, the authors developed a sequential treatment strategy in which Y27632 was administered during the early post-injury phase, followed by CHIR99021 and finally SB431542. This combined and sequential strategy resulted in a more complete recovery of tissue function, with improved calcium signal propagation and reduced fibrotic remodelling.

The efficacy of this strategy was further confirmed in a murine model of myocardial infarction, in which sequential treatment reduced the fibrotic area and improved cardiac function compared with treatment with the individual modulators [30].

In vivo validation thus supported the ability of the 3D model to reproduce post-injury remodelling. It also highlighted its potential as a screening platform for identifying pharmacological strategies that may prove effective in vivo.

Biomimetic platforms are progressively integrating sensing technologies, enabling continuous, non-invasive monitoring of tissue mechanics and cellular responses in real time. A seminal example has been the development of a force-sensing pulmonary fibrosis-on-a-chip, in which the biomimetic platform itself functions as a mechanical sensor. Unlike conventional approaches, this system enables continuous quantification of tissue contraction and stiffness during fibrosis progression, directly correlating the molecular activation of profibrotic programs with tissue biomechanical remodelling and pharmacological responses [44]. This strategy further expands the capabilities of biomimetic platforms by enabling direct and continuous monitoring of tissue biomechanical remodelling throughout disease progression.

Building upon this concept, a subsequent study translated the same principle to cardiac fibrosis and repair by integrating an optical nanosensor directly within a three-dimensional engineered tissue. Tissue deformation was thereby converted into an optical signal, enabling real-time visualisation of biomechanical remodelling during fibrosis progression while simultaneously supporting the screening of multiple antifibrotic compounds [45].

### 3.2. From Mechanobiology to Assay Design

The mechanisms described above have direct consequences for how antifibrotic assays should be designed and interpreted. Standard tissue-culture plastic is orders of magnitude stiffer than healthy (~10 kPa) or fibrotic myocardium, so fibroblasts cultured on it are already mechanically pre-activated. This ceiling effect can mask the activity of compounds that act upstream on mechanosensing, producing false negatives. Matrices with tunable stiffness covering the physiological-to-fibrotic range [31,32] allow drugs to be tested both in preventing activation (soft-to-stiff) and in reversing it (stiff-to-soft), a distinction relevant to patients who are treated when fibrosis is already established. In addition, the mechanical regime should match the disease being modelled. Cyclic stretch [23] reproduces the load experienced by fibroblasts in overloaded myocardium, whereas local injury models [30] capture the regional stiffness gradients of a post-infarction scar. Importantly, the readouts should ideally include mechanical and functional endpoints (tissue stiffness, passive tension, contractile force), in addition to molecular markers, because the ultimate aim of antifibrotic therapy is to restore tissue mechanics and function. Notably, the mechanosensitive pathways are themselves druggable (Table 3). Testing compounds that target these pathways in a mechanically inappropriate context may underestimate their efficacy, whereas testing them in a mechanically matched context may also reveal on-target effects on cardiomyocytes that are relevant to safety.

## 4. Current Limitations, Benchmarks and Standardisation

### 4.1. Biological and Technical Limitations

A critical assessment of the studies reviewed here points to several biological and technical limitations that should be considered when interpreting their findings. The main ones are listed below.

Cell immaturity. hiPSC-derived cardiomyocytes and fibroblasts retain a largely fetal-like phenotype. Physical conditioning can advance maturation [46], but electromechanical properties remain below those of adult myocardium. Fibrosis develops mainly in adult and ageing hearts, so immature cells may respond differently to both profibrotic stimuli and drugs.

Species, donor and line variability. Several platforms still use rodent cells [20,22,31,32], which limits their value as human-relevant systems. In human-based studies, many platforms rely on a single donor or cell line, e.g., a single commercial donor of atrial fibroblasts [23]. Cardiac fibroblasts from different donors, chambers (atrial vs. ventricular) and disease states differ in their baseline activation, and hiPSC lines vary in differentiation efficiency. Testing across several donors and lines is therefore needed to tell general drug effects from donor-specific ones.

Missing cell types. Most platforms focus on the main cardiac cell types and lack immune cells, although macrophages are known key regulators of cardiac fibrosis and can mediate as well as determine drug responses [20]. Also, endothelial cells and perfusion are often absent, so hypoxia gradients, inflammatory cell recruitment and realistic drug delivery cannot be reproduced.

Matrix variability. Naturally derived matrices (matrigel, collagen and fibrin from different sources) vary from batch to batch in composition and stiffness, which directly affects fibroblast activation. Synthetic and decellularised matrices offer better control but may lack relevant biochemical cues.

Fibrosis induction, time scale and exposure. Exogenous TGF-β, hypoxia, stretch, cryoinjury and an increased fibroblast fraction produce different fibrotic phenotypes, and none fully reproduces the months-to-years development of human fibrosis. Most cultures last days to a few weeks, which limits the study of chronic remodelling and of long-term drug effects. Drug concentrations are rarely matched to clinically relevant free plasma levels, and there is no metabolism or systemic exposure.

### 4.2. What Would Demonstrate Predictive Superiority?

Showing that a 3D model is more predictive than simpler systems requires more than showing that it is more physiological. In our view, a direct comparison, whereby the same compound set is tested in parallel, under matched conditions, in the 3D platform and in conventional 2D assays, would be optimal, and this approach has indeed been adopted [20]. The inclusion of reference compound panels with known outcomes (e.g., compounds with established antifibrotic efficacy in vivo, compounds that failed in vivo or in clinical trials, and inactive or cytotoxic controls) would allow a more precise calculation of sensitivity, specificity and predictive values, which are currently inferred from only a few positive examples. In terms of increased translational potential, the study design would benefit from clinically relevant exposure and endpoints, with treatment concentrations matched to human free plasma levels, endpoints related to clinically measurable outcomes (tissue stiffness, diastolic function, collagen turnover markers), and treatment performance confirmed across donors, cell lines and independent laboratories. Ultimately, retrospective benchmarking against drugs with known clinical outcomes in cardiac fibrosis or heart failure (e.g., renin–angiotensin–aldosterone system inhibitors, SGLT2 inhibitors, pirfenidone, which has been tested in HFpEF) should be used to approach validation in human patient cohorts. In the randomised, double-blind, placebo-controlled phase 2 PIROUETTE trial (NCT02932566), completed in 2020, 52 weeks of pirfenidone in patients with HFpEF and myocardial fibrosis reduced myocardial extracellular volume, measured by cardiovascular magnetic resonance, compared with placebo (between-group difference, −1.21%), meeting the primary outcome; these favourable effects, however, remain to be confirmed in larger trials [47]. An example of this type of approach has been offered by the cardiac safety field, where hiPSC-cardiomyocyte assays for proarrhythmic risk were qualified through a blinded, international multisite study of 28 drugs with known clinical risk, which also quantified the effects of cell line, site and platform [48].

### 4.3. Towards Standardisation

The studies reviewed here use different fibrosis triggers, endpoints and comparators, which makes cross-platform comparison and routine adoption particularly difficult. We propose that future studies adopt a minimal common framework, whereby every study should include at least one positive control acting on the TGF-β axis (e.g., a TGF-β receptor inhibitor such as SB431542, already used in several platforms [20,30]), a clinically used antifibrotic drug as comparator (e.g., pirfenidone, which is approved for idiopathic pulmonary fibrosis but remains investigational in cardiac fibrosis, and is the most frequently tested compound in the platforms reviewed [16,19,23]), and a vehicle/inactive control. Cytotoxicity, myofibroblast activation (α-SMA), collagen deposition and/or a collagen turnover marker, together with at least one mechanical endpoint (stiffness or passive tension) and, when cardiomyocytes are present, contractile force, should always be reported, also to exclude non-specific effects. Blinded studies across several laboratories using shared protocols, cell lines and compound sets, following precedents such as the Comprehensive in vitro Proarrhythmia Assay (CiPA) hiPSC-cardiomyocyte multisite study [48] and the context-of-use framework proposed by the IQ Microphysiological Systems Affiliate [49], would allow the necessary multicentre validation of these platforms.

## 5. Conclusions and Future Perspectives

The heart represents one of the most paradigmatic examples of a system continuously operating within a highly dynamic environment [33]. Its physiological function relies on a finely tuned balance between mechanical stimuli and cellular responses.

Cardiac fibrosis is now increasingly recognised as a self-sustaining repair process in which biomechanical remodelling is no longer regarded as a mere consequence of disease progression, but rather as an active contributor to its perpetuation. Following cardiac injury, fibroblast activation, extracellular matrix remodelling and altered tissue mechanics progressively reshape the cardiac microenvironment, establishing reciprocal feedback mechanisms that sustain fibrotic progression. Importantly, this process does not involve fibroblasts in isolation, but emerges from the coordinated interplay among multiple cell populations, the extracellular matrix and the surrounding biomechanical environment [33,34,39].

The recognition that cardiac fibrosis is driven by reciprocal interactions between cells and their mechanical microenvironment has inevitably influenced the way this reparative process is experimentally modelled. Conventional two-dimensional culture systems are intrinsically unable to reproduce the spatial organisation of tissues, extracellular matrix architecture, dynamic mechanical properties and the multicellular interactions governing mechanobiological signalling in vivo. Consequently, advanced three-dimensional models have progressively emerged as physiologically relevant platforms for recapitulating these complex cell–matrix interactions, providing improved systems for investigating the pathogenic mechanisms of fibrosis and evaluating novel antifibrotic therapeutic strategies [15,34].

In recent years, however, these models have paved the way for a new phase in experimental modelling, aimed not only at reproducing the pathological microenvironment but also at generating increasingly informative functional readouts. Recent advances in organ-on-a-chip engineering are already moving in this direction [50].

Overall, current evidence indicates that multicellular three-dimensional models are progressively expanding the role of preclinical platforms, moving beyond experimental validation toward systems capable of supporting the identification, selection and repurposing of antifibrotic candidates. In this perspective, one of the major future challenges will not simply lie in developing increasingly advanced three-dimensional models, but in designing platforms capable of generating more informative functional as well as mechanistic readouts. By providing a more comprehensive and dynamic assessment of therapeutic responses, such systems may improve candidate prioritisation, although this potential has so far been supported by a limited number of studies and still needs to be demonstrated systematically.

Beyond improving the efficiency and predictive value of preclinical drug screening, the increasing availability of advanced human-based models is also consistent with the current regulatory shift toward New Approach Methodologies (NAMs). This shift is driven not only by ethical considerations and the principles of the 3Rs (Replacement, Reduction and Refinement), but also by the recognised translational limitations of animal models. Although animal studies remain important in basic and preclinical research, their predictive value is constrained by interspecies differences and by their limited ability to reproduce human physiological, pathological and therapeutic responses [51]. In this direction, both the FDA and EMA are progressively promoting the incorporation of NAMs into drug development and regulatory assessment. These approaches can complement conventional animal testing and, where appropriate, reduce its use by providing more human-relevant in vitro and computational evidence.

In this framework, human-based three-dimensional models complement animal experimentation by providing tissue-specific pharmacodynamic information before in vivo validation, while animal studies would be reserved for systemic aspects that cannot yet be fully recapitulated in vitro, such as pharmacokinetics, biodistribution and whole-organism safety.

Ultimately, achieving these goals will require closer integration of cell biology, mechanobiology and bioengineering to develop experimental platforms equipped with sensing technologies capable of providing continuous structural, functional and biomechanical readouts beyond static endpoint analyses. Combined with rigorous validation and standardisation, such an evolution could transform three-dimensional models from tools used primarily to recapitulate cardiac fibrosis into validated platforms that support drug discovery and improve preclinical translation for enhancing cardiac repair therapies.

## Figures and Tables

**Table 3:** Mechanosensitive mechanisms and their implications for antifibrotic assay design.

Mechanism	Evidence in Cardiac Models	Supporting Non-Cardiac Evidence	Implications for Assay Design	Pharmacological Tools/Targets	Impact on Interpretation of Drug Responses
Matrix stiffness—integrin β1/FAK	3D collagen–alginate hydrogels with dynamic stiffening/softening in neonatal rat CFs [31]; FAK–NF-Y control of CF proliferation [37]	—	Use tunable matrices spanning healthy to fibrotic stiffness; test both prevention and reversal	FAK inhibitors; integrin-blocking strategies	On rigid substrates, ceiling effect may hide activity (false negatives)
YAP/TAZ–TEAD	3D cardiac fibroblast cultures [38]; stiffness-dependent YAP nuclear localisation and 98-gene mechanosensitive signature in 3D [32]	General mechanobiology [36]	Report YAP/TAZ localisation as a mechanistic readout; consider spatial heterogeneity within 3D constructs [38]	YAP–TEAD interference [38]	Spatial position within the construct changes the response; 2D cultures may overestimate homogeneous activation
MRTF/SRF	Mostly inferred; limited direct cardiac 3D evidence [36]	Multiple fibrotic tissues [35]	Combine with stiffness and stretch control	MRTF/SRF pathway inhibitors	Mechanistic attribution in cardiac fibroblasts still requires direct validation
Rho/ROCK and cytoskeletal tension	hEHT injury–repair model: early ROCK inhibition (Y27632) partially rescued remodelling; best effect in sequential combination [30]	—	Requires models with temporal resolution of remodelling phases	ROCK inhibitors (Y27632)	Efficacy is phase-dependent; single-time-point assays may miss it
Piezo1 and TRP channels	Piezo1 functional in human and mouse cardiac fibroblasts, coupling to IL-6 secretion [41]; Piezo1 in heart mechano-chemo transduction in vivo [40]	Adipose-derived stem cells on fibrotic-like nanostructured scaffolds [42]; skin fibrosis collagen lattices [43]	Matrix architecture/fibre alignment, not only stiffness, should be controlled	Piezo1 agonist Yoda1; blockers (e.g., GsMTx4, Gd3+); TRP modulators	Channel also expressed in cardiomyocytes and endothelial cells: multicellular models are needed to assess effects in these cell types, which may reflect on-target activity rather than off-target effects
Cyclic stretch/mechanical load	uScar: stretch alone induced fibrosis and limited miRcombo efficacy [23]	Force-sensing lung fibrosis-on-a-chip [44]	Match strain amplitude and frequency to the disease being modelled	Load-modulating drugs; mechanosensor inhibitors	Therapies effective under static conditions may lose efficacy under load (false positives in static assays)

Abbreviations: CF, cardiac fibroblast; FAK, focal adhesion kinase; hEHT, human-engineered heart tissue.

## Data Availability

No new data were created or analyzed in this study. Data sharing is not applicable to this article.
